# Container-based sanitation: assessing costs and effectiveness of excreta management in Cap Haitien, Haiti

**DOI:** 10.1177/0956247815572746

**Published:** 2015-04

**Authors:** Sebastien Tilmans, Kory Russel, Rachel Sklar, Leah Page, Sasha Kramer, Jennifer Davis

**Affiliations:** e-mail: stilmans@stanford.edu; e-mail: kcrussel@stanford.edu; e-mail: rsklar@berkeley.edu; e-mail: lnevada@oursoil.org; e-mail: skramer@oursoil.org; Department of Civil & Environmental Engineering, Stanford University, California, USA, e-mail: jennadavis@stanford.edu

**Keywords:** container-based sanitation, faecal management, urban sanitation, waste infrastructure, waterless sanitation

## Abstract

Container-based sanitation (CBS) – in which wastes are captured in sealable containers that are then transported to treatment facilities – is an alternative sanitation option in urban areas where on-site sanitation and sewerage are infeasible. This paper presents the results of a pilot household CBS service in Cap Haitien, Haiti. We quantify the excreta generated weekly in a dense urban slum,^(1)^ the proportion safely removed via container-based public and household toilets, and the costs associated with these systems. The CBS service yielded an approximately 3.5-fold decrease in the unmanaged share of faeces produced, and nearly eliminated the reported use of open defecation and “flying toilets” among service recipients. The costs of this pilot small-scale service were higher than those of large-scale waterborne sewerage, but economies of scale have the potential to reduce CBS costs over time. The paper concludes with a discussion of planning and policy implications of incorporating CBS into the menu of sanitation options for rapidly growing cities.

**Figure:**
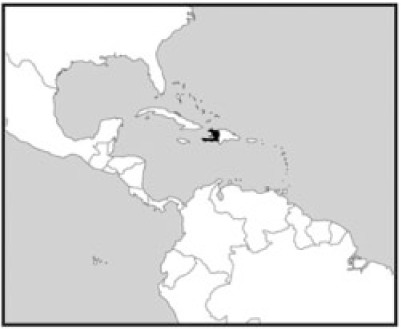


## I. Introduction

The UN Millennium Development Goals (MDGs) process has helped to raise public awareness of the substantial gap in access to even the most basic of sanitation services within developing regions. Current estimates suggest that 2.5 billion people, 35 per cent of the global population, will still lack access to improved sanitation services at the close of the MDG process in 2015.^(2)^ Thirty per cent of these people will live in urban households, primarily in sub-Saharan Africa and South Asia.^(3)^ Indeed, over the next 35 years, there will be an additional 2.4 billion people in cities of low- and middle-income countries, representing faster growth rates than ever before seen, particularly for sub-Saharan Africa.^(4)^ Such rapid population growth is likely to aggravate the challenges of sanitation planning in urban settings.

Conventional sewerage is unlikely to be the prevailing paradigm for sanitation investments in these regions for the foreseeable future.^(5)^ Sewer systems require considerable up-front capital investment, and they depend on the availability of reliable water and energy supplies for moving excreta through the sewer network.^(6)^ Sewer networks also depend upon highly professional, well-resourced utilities to operate and maintain them. Finally, sewers can be politically challenging to install, as extending networked infrastructure to informal settlements can confer legitimacy on the illegal occupation of land, disrupting the integrity of property laws.^(7)^ Against these challenges and the rapid pace of urban growth, the share of the urban population with sewer connections in least developed countries fell from an estimated 12 per cent in 1990 to 11 per cent in 2010.^(8)^

Among those urban residents who do have access to improved sanitation facilities in Africa and Asia, the large majority use on-site solutions such as pit latrines and pour-flush toilets connected to septic tanks.^(9)^ From an urban planning perspective, the fact that responsibility for financing and maintaining such facilities typically falls to households carries both advantages and disadvantages. Mobilizing private resources for household sanitation eliminates (or delays) the need for municipal governments to make major investments in trunk (main) sewer infrastructure. However, since a large proportion of residents of urban slums are renters,^(10)^ they rely on landlords who may have limited incentives to invest in sanitation.^(11)^ Also, households are more likely to invest in the aspects of sanitation that confer such individual benefits as convenience, privacy and prestige, while under-investing in the conveyance and treatment systems that are critical for realizing public health and environmental benefits.^(12)^

In many urban areas, density and poverty levels are such that shared or communal facilities are viewed as the only viable sanitation alternative.^(13)^ However, there is an enduring debate about whether shared facilities can be reliably hygienic and accessible enough (especially at night) to deliver the intended public health and well-being objectives.^(14)^

The conditions in many urban areas hamper the success of both private and shared on-site facilities. The high transience of residents impedes investment in private facilities and reduces residents’ bargaining power with landlords and authorities.^(15)^ It also disrupts the social networks that would be conducive to maintenance of shared facilities.^(16)^ Low sewer penetration and poor drainage in many cities mean that shared and private facilities alike often face clogging and flooding issues,^(17)^ and rely on pit and septic tank emptying services for managing an estimated 876 million tonnes of faecal sludge each year.^(18)^ In many instances, emptying must be performed manually, resulting in substantial faecal exposure for both service operators and the general public. A large practitioner literature suggests that the public health goals commonly ascribed to sanitation are often not met by this largely informal and unregulated market.^(19)^ There remains a pressing need for service alternatives that are better adapted to the challenges of dense, low-income urban areas.

Over the past several years, a small number of organizations have begun experimenting with container-based sanitation (CBS) systems as an alternative model for excreta management. A typical CBS system includes a toilet that captures waste not in a deep pit or a septic tank, but in a container that can be easily sealed and removed from underneath the pedestal or squat plate. CBS systems are typically waterless; most rely on urine-diverting toilets that use dry cover material, chemicals, or biodegradable plastic film for odor and pest control. Waste containers are sealed and transported to centralized facilities for cleaning, and the waste is either treated and discharged, or processed to recover resources like energy, nutrients or water. CBS systems present several potential advantages over on-site systems: the sealed vessels can be transported without releases to the environment; the systems are compact because space for excreta storage is minimized; the systems can be movable to more readily accommodate the needs of renters or transient residents; and finally, they incorporate by design an end-to-end containment, removal and management strategy for faeces.

The image of a CBS system can bring to mind related, but quite different, models of excreta management such as manual scavenging and “bucket latrines” or “pail systems”. While pail systems were originally deployed in cities as a perceived improvement upon pit latrines,^(20)^ these approaches have been the subject of vigorous opposition on moral and public health grounds, as well as the lack of labourers willing to operate them.^(21)^ In Kampala, Uganda, the “single-bucket” system was considered labour-intensive and unhygienic, in part because emptying of buckets into streetcarts resulted in frequent spills.^(22)^ Likewise, bucket systems were phased out in Kisumu, Kenya, because of *“the health risks, disposal problems and lack of social acceptability”*.^(23)^ Bucket latrines are being phased out in Kumasi, Ghana, because they are emptied by unlicensed operators into unsanitary locations like streams or bushes.^(24)^ A CBS system shares with these models the feature of manual collection of excreta in relatively small containers. Importantly, however, CBS systems incorporate measures to isolate excreta from human contact throughout the supply chain of storage, transport and disposal. In this manner CBS systems are modern evolutions of the “earth closet” systems first developed by Henry Moule and colleagues in the mid-1800s, in which faeces were captured in containers and covered with dry earth.^(25)^ Historically, this system was perceived to be preferable to pail systems and privies or cesspits, and in some cases even to waterborne sewerage.^(26)^

In this study, we present the results of a pilot CBS service providing household sanitation services for low-income households in an informal community of Haiti. These households previously relied on several high-quality public toilets, as well as some private pit toilets, for their sanitation needs. We pursue the following questions: How does the provision of a household CBS option change household sanitation behaviours and the share of the excreta produced in the community that is safely managed to meet public health objectives? How do the capital and operating costs of a household CBS system compare to those of leading alternatives? What might be the planning implications of including CBS systems on the sanitation menu of options for hard-to-serve urban areas?

Following this introduction, we describe the study site and the features of the CBS service provided to participants. In Section III, we describe our data collection and analysis approach. Section IV presents findings regarding demographic attributes of sample households, changes in household sanitation behaviours, and the share of sample household excreta produced that is safely managed following the CBS intervention. In Section V, we compare unit costs for the household CBS system and those for public toilets, a common strategy for sanitation service provision in low-income urban communities. We discuss the planning and policy implications of including household CBS systems as a service option in Section VI, as well as the research still needed to explore the potential and viability of CBS.

## II. Study Site and Service Description

Shada is an informal community of roughly 9,300 residents, covering approximately 7 hectares near the centre of Cap Haitien, Haiti. A network of alleys crisscrosses the community, ranging in width from 0.4 to 1.5 metres. Shada is practically at sea level; the water table is about 1 metre below the ground surface, and stormwater drainage canals have raised walls in the lower areas to help channel runoff to the river. Floods in Shada are common, and the community experienced several during the development and implementation of this study. Because of the frequent flooding and the narrow alleys that preclude hygienic emptying, pit latrines in this community are an inadequate sanitation option.

The non-governmental organization Sustainable Organic Integrated Livelihoods (SOIL) operates three container-based public toilet blocks in Shada, which are free for anyone to use. Seventy per cent of Shada households live within 100 metres of these blocks, and the farthest household is located about 220 metres from the nearest block. Each toilet block has a paid attendant supervising two urine-diverting stalls for adults and one smaller, non-diverting stall for children. Users or the attendants throw dry cover material, typically sugarcane bagasse (a fibrous byproduct of sugarcane crushing), into the container after each use to cover the faeces. Sixty-litre and 20-litre containers are used for faeces and urine collection, respectively. The toilets are typically open from 5:30 until 22:00, but they often close earlier or later at night depending on user traffic. All full containers from the public toilets are sealed, carried to the roadway, and trucked by SOIL’s team to its compost site 16 kilometres away. Containers are emptied into bins where the waste is composted thermophilically.^(27)^ Containers are then power-washed and soaked in a chlorine bath before being stored for reuse. Clean containers containing fresh bagasse are returned to the public toilets.

### a. Household toilet service deployment

Between September 2011 and August 2012, a low-cost, urine-diverting, container-based toilet suitable for households in Shada was developed through a user-centric design^(28)^ process. The toilet is a portable box-shaped (38 centimetres width x 48 centimetres length x 46 centimetres height) pedestal built around two sealable containers. A 20-litre container captures faeces, and users cover the faeces with dry cover material (“Bonzodè”, a mix of sieved sugarcane bagasse and crushed peanut shells) after each use. Urine is captured in a 3.8-litre container that users empty as necessary in canals, the sea, or soakaway pits. The toilet also features a white, western-style toilet seat. Toilets were distributed in November 2012 to 135 households in 30 randomly selected clusters for a three-month free service pilot. Users were told that they would have to start paying a monthly subscription fee at the end of the free trial period, or to return their toilet at no cost.

For the duration of this trial, SOIL operators used specially-designed carts to collect waste containers from each household twice weekly. Each full container was exchanged for a clean container holding a fresh supply of Bonzodè; it was then transported by cart to the road, and by truck to the compost site. Clean containers were returned to Shada, and were filled with Bonzodè before being returned to circulation in the service. At all times, each household had two containers: one in use in the toilet, and one containing fresh Bonzodè.

## III. Methods

The 135 Shada households that agreed to participate in the household service trial, along with 151 randomly selected Shada households not involved in the service, completed in-person interviews in October 2012 (before toilet installation). In February 2013, after three months of service, 127 households participating in the service trial and 115 non-participating households were interviewed again. Respondents were asked to identify the sanitation option that men and women in their household used for defecation during the day and at night; how frequently respondents had used that option in the day prior to the interview; and the frequency and timing of their defecation.^(29)^ Respondents with children under 5 years old were also asked how they typically disposed of their children’s faeces.

### a. Service monitoring

A container and weight tracking system was developed for the CBS service. SOIL staff recorded the number and weight of each toilet container arriving at the compost site, as well as the weight of each bagasse container and sack of Bonzodè leaving the compost site for the public toilets and household toilets, respectively. Service monitoring began in August 2012 for the public toilets, and continued for 30 weeks. Monitoring of the household service began in November 2012 for 17 weeks (including four weeks of startup).

Observations were also conducted at SOIL’s three public toilets in Shada for a total of five days each (three during the period August–October 2012 and two in February–March 2013). A Haitian observer noted the gender of each toilet user and identified each user as an adult or child without asking for the age directly. The observer also asked each user if s/he had used the toilet to defecate or only to urinate. The observer weighed each stall’s faeces container and cover material receptacle at the beginning and end of each observation day, and each time a full faeces container was replaced with an empty one. Observations were typically conducted between 6:00 and 20:00.

### b. Share of generated faeces that is safely managed

The mass of a single defecation was computed using the public toilet observation data. For each adult stall in the public toilets,^(30)^ the mass of cover material used over the course of the observation day was subtracted from the total mass of material accumulated in the waste container to determine the mass of faeces collected in each stall. For each observation day, the total mass of faeces was divided by the total number of defecations to determine the mean weight of one defecation. The mean of the values from all observation days, weighted by the number of defecations in each day, was calculated as the mean mass of a single defecation.

Using this mean mass value and survey respondents’ reported defecation practices, the total mass of faeces managed through each sanitation alternative was calculated using Equation (1) and Equation (2).

(1) $$M_{faeces}=M_{defecation}*F_{defecation}*P_{Age>5}$$

(2) $$M_{i}=M_{faeces}*[(f_{day}*f_{i,day})+(f_{night}*f_{i,night})]$$

Where:

*M_faeces_* = Total mass of faeces produced in the cohort (kilogram)

*M_defecation_* = Mass of one defecation (kilogram/defecation)

*F_defecation_* = Daily frequency of defecation (defecations/person/day)

*P_Age>5_* = Cohort population older than 5

*M_i_* = Mass of faeces disposed in a sanitation option *i*

*f_day_, f_night_* = Fraction of defecations occurring by day or night

*f_i,day_* = Fraction of population using sanitation *i* option *by day*

*f_i,night_* = Fraction of population using sanitation option *i at night*

Excreta deposited in the public and the pilot household toilets were considered to be safely managed, because the toilets are coupled with a mechanism for waste collection and treatment. Excreta deposited in pit latrines were not, because no viable containment or management system exists for them in Shada. It was assumed that each respondent accurately identified the sanitation option used by the other adults in their household; that all adults of the same gender in a household followed the same habits; and that children of age 5–17 followed the same defecation habits reported for the adults of their gender in the household. Faeces management for children younger than age 5 was considered safe if a respondent reported disposing of faeces in the public toilets or the household CBS toilets.^(31)^

For verification, the calculated mass of waste produced by users of the household toilet service was compared to the measured mass of faeces collected. Each week, the mass of cover material delivered to the toilets was subtracted from the mass of material arriving at the compost site from the toilets, yielding the net mass of faeces that was safely removed from the community.^(32)^ We assume that there is a negligible mass of materials in the containers other than faeces and cover material.

### c. Evaluating service costs

SOIL’s weekly expense reports and historical capital expense data were used to calculate the costs of establishing and operating the two waste conveyance systems–public and household CBS services. These costs are reported for the 13-week period during which the household CBS service had reached its full planned operating level. Costs are expressed in absolute terms, but also as a unit cost per kilogram of faeces managed. Direct costs for labour; employee benefits (food, transport, and communication stipends); supplies and repairs; and consumables (chemicals, cover material, toilet paper) were itemized. Management staff salaries and vehicle expenses were apportioned to each service according to SOIL’s estimates of the allocation of these resources during operations. General organizational costs like office rental were apportioned equally among all of SOIL’s projects. All costs were also categorized as fixed (costs incurred regardless of the level of activity of each service, such as manager salaries) or variable (costs that change with the scale of operation, such as waste collector payments). Costs for sewerage in low- and middle-income contexts were obtained from published literature.

## IV. Defecation Practices and Faeces Management

**Figure 1:**
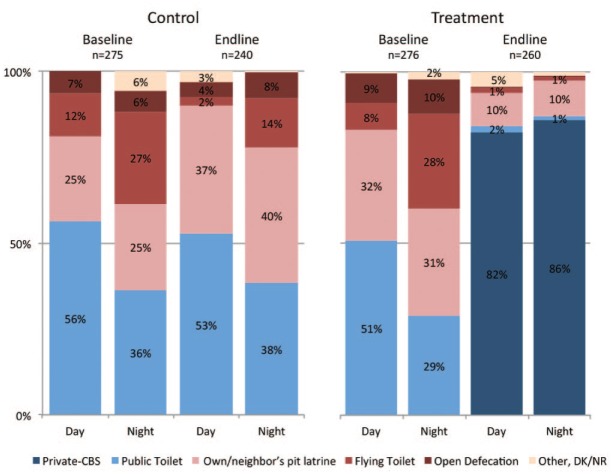
Reported facilities used by adults to defecate by day and by night NOTE: DK = “Don’t know”; NR = “No response”

The sanitation options that sample households reported using to defecate by day and by night are shown in Figure 1. At baseline, approximately half of households used the public toilets during the day, but only one third used them at night. Instead, respondents reported higher use of flying toilets^(33)^ and open defecation at night. Defecation practices in the control cohort remained similar between baseline and endline, aside from a reported increase in the use of pit latrines.^(34)^ In the treatment cohort, the household CBS service nearly eliminated reported open defecation and use of flying toilets at endline. In contrast to use of public toilets, rates of household toilet use were comparable during day and night.

### a. Faeces produced and share of faeces managed

During the observation period, the three public toilets in Shada together served approximately 1,364 users between 6:00 and 20:00. A new user entered each stall on average every 5.5 minutes.^(35)^ Twenty-five per cent of users were adult men, 37 per cent adult women, and 38 per cent children. Ninety-seven per cent of users were Shada residents. Almost no users (0.3 per cent) reported using the toilets only to urinate. The weighted mean mass of an adult defecation was 163 grams (standard deviation of 14 grams).^(36)^ SOIL collected approximately 1,524 kilograms of faeces per week from the public toilets during the study period, or 1.12 kilograms per user per week. Additional information on waste collection from public toilets is provided in the supporting information.

**Figure 2:**
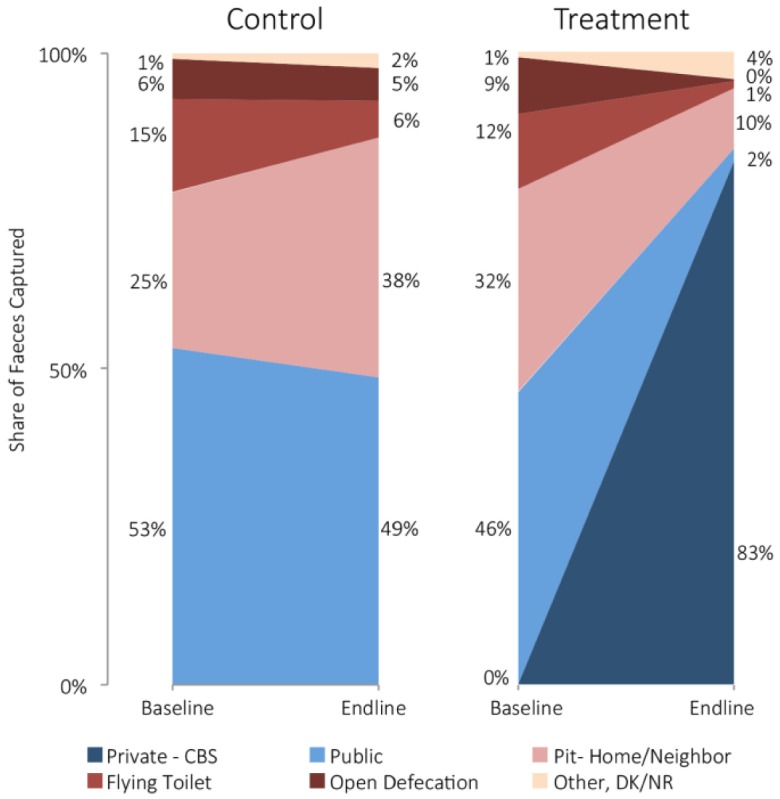
Share of faeces captured by sanitation practice, cohort and study phase

The share of each cohort’s waste disposed of via each sanitation alternative is shown in Figure 2. The median reported frequency of defecation was once per day, with four fifths occurring by day at both baseline and endline. The proportion of the treatment cohort’s waste that was safely managed increased from 46 per cent at baseline to 85 per cent at endline, whereas the proportion of managed faeces in the control cohort decreased slightly from 53 per cent to 49 per cent. Notably, the proportion of waste reportedly disposed of via open defecation and flying toilets in the treatment cohort dropped to less than 2 per cent.

The total calculated mass of faeces deposited in the CBS household toilets was approximately 613 kilograms per week.^(37)^ Weight data from the waste collection service corroborate this estimate (Table 1). Indeed, the mean weekly mass of faeces collected through the household service, 652 kilograms, suggests that the CBS service may be capturing more waste than that produced by the households who received the toilets (i.e., that visitors and/or neighbours may be using the toilets).

**Table 1:** Weekly waste collection from household CBS at full deployment (13 weeks)

	Collected Material	Delivered Cover Material	Net Faeces Collected
Mean (kg)	943	291	652
Standard deviation (kg)	96	106	164
Max (kg)	1,108	416	1,000
Min (kg)	800	99	419
Per user^(1)^ (kg)	1.79	0.55	1.24

NOTE:

(1) Based on 526 household CBS users above the age of 5.

Reported safe management of infant faeces also increased in the treatment cohort from 12 per cent at baseline to 49 per cent after deployment of the household service pilot. By contrast, the share of safely managed infant faeces reported in the control cohort changed from 6 per cent to 4 per cent between baseline and endline. The primary alternative for disposal of infant faeces was the waterway near Shada.

There is evidence that occasional unintended releases of faeces occurred from the CBS service into the environment. Sixteen respondents (15 per cent) in the treatment cohort reported that their household was missed during a collection day, for a reported total of about 16 missed collections out of the approximately 3,300 intended collections over 17 weeks of service.^(38)^ Two respondents (13 per cent of those whose houses were missed) reported emptying their toilet in a canal or the river after a missed collection, 10 respondents (63 per cent) reported waiting until the next collection, two carried the container to SOIL’s public toilet for replacement, and two did not report what recourse they took. Assuming the two reported incidents of waste dumping were the only discharges from the household CBS system, they represent less than 0.5 per cent of the total excreta removed by the household service over the duration of the study period. There was also one reported instance of a toilet being stolen by a respondent’s former partner. It was impossible to determine where the toilet was taken, how it continued to be used, or its resulting impact on the management of waste produced by the thief. The respondent reverted to pre-existing defecation habits.

Container filling and availability of cover material also affected toilet use. Fifteen respondents (14 per cent) reported at least one instance of their container being filled before collection day. Seven of them stopped using the toilet until the next collection, while the remainder sealed the container and either replaced it with their spare container or brought it to the service collection depot for replacement. Thirty-one respondents in the treatment cohort (28 per cent) reported running out of Bonzodè at least once during the pilot service. Although 17 of those obtained some extra material from neighbours or from the household collection team before collection day, 13 ceased using the toilet until the subsequent service,^(39)^ and one used the toilet without Bonzodè.

## V. Service Costs

The total and unit costs of both the public and household toilet CBS services are summarized in Table 2. At this early stage in the development and deployment of these services, operating costs represent a high proportion of total costs. Labour, primarily management, constitutes a major share of costs for all services. Vehicle costs are also significant for both services. Important consumables for the public toilets include cleaning products and toilet paper (accounting for 23 per cent of public toilet variable costs), whereas preparation of Bonzodè (grinding peanut shells) accounts for nearly all consumables costs in the household service (and 37 per cent of all household CBS variable cost). The collection and conveyance cost of the household CBS service was approximately US$ 22/household/month.

**Table 2:** Costs of container-based waste collection and treatment (13 weeks)

	CBS Conveyance	
	Public	Household
Total Capital Costs:	US$ 24,148	US$ 18,742
Construction, Installation:	70%	67%
Land:	0%^(1)^	0%
Vehicles:	30%	33%
		
Total Operating Costs:	US$ 10,602	US$ 7,057
Labour:	51%	40%
Benefits:	6%	13%
Facilities Maintenance:	3%	6%
Vehicles & Equipment:	21%	23%
Consumables:	14%	12%
Overhead:	4%	6%
Percentage Fixed Costs:	44%	71%
Amortized Unit Capital Costs (US$/kg of faeces):	US$ 0.12	US$ 0.13
Unit Operating Costs (US$/kg of faeces):	US$ 0.54	US$ 0.83
Total Unit Cost (US$/kg of faeces):	US$ 0.66	US$ 0.96

NOTE: ^(1)^The land for the public toilets in Shada was donated by local residents.

## VI. Discussion

The provision of the household CBS service in Shada virtually eliminated reported open defecation and use of flying toilets, triggered a 4-fold increase in safe management of infant faeces, and yielded a 3.5-fold reduction in the share of unmanaged faeces in the treatment cohort. Despite the fact that the public toilets available to households in the study area are free to use, open long hours, and kept clean by a professional attendant, many residents still practise open defecation or use flying toilets to meet their needs after dark. Household sanitation offers the opportunity to achieve high rates of faeces management both day and night, and improve management of infant faeces.

Nevertheless, the unit cost of household CBS service was higher than that of the public CBS facilities. The capital costs of the public toilets may be underestimated because the land for the toilet sites was donated. In addition, we believe that the costs of the household service may represent a high estimate for two reasons. First, as a pilot this service did not reflect cost-saving measures that SOIL subsequently identified and continues to implement, including lower-cost toilets and streamlined collection procedures. Second, unit costs in the pilot could not exploit potential economies of scale. In particular, the direct labour costs of the household service (transporting waste from households to the compost site), which should increase with increasing service area, constituted only 12 per cent of the total labour costs in the pilot. The remaining labour costs – largely managerial and outreach tasks – should enjoy considerable economies of scale over the longer term. In contrast, the public CBS is less likely to benefit from economies of scale, because the toilet attendants constitute the majority (55 per cent) of labour costs. Such labour costs will generally increase proportionally with increasing coverage area and number of toilets deployed, although the number of stalls per attendant could potentially be increased as a cost-saving measure.

More generally, the high proportion of fixed to total costs in both the household and public CBS service is to be expected in early-stage enterprises. The continued experimentation and iteration involved in optimizing the services require substantial input from managers and planners, while the services operate below their intended capacities. Such experimentation is important to identify and exploit opportunities for improving service quality and efficiency. As one example, the pilot illuminated the importance of improving the Bonzodè procurement process (e.g., through processing improvements or substitution with a cheaper alternative) for reducing costs of the household CBS service. Pilot work is thus essential for determining the potential for exploiting economies of scale in a household CBS service, and the factors on which those economies depend.

At the current scale, the unit cost of the household CBS service in Shada is US$ 0.96 per kilogram of faeces, considerably higher than costs reported for condominial^(40)^ and conventional sewerage in the literature. Amortized costs of piped sewer systems range between US$ 0.14 and 0.21/kilogram of faeces for low-cost (condominial) sewerage and US$ 0.30 and 0.46/kilogram of faeces for conventional sewerage (see this paper’s supporting information online).^(41)^ Importantly, the sewer cost data do not include the costs of piped water supply, which is typically necessary for a sewer system to function properly and may double the cost of sewer service.^(42)^ On the other hand, the costs of urine disposal are not included in the CBS service estimate, because households in Shada assumed responsibility for this function.

It is also important to note that the costs of waste treatment are not included in this cost analysis. Further research is necessary to evaluate the relative costs of treating excreta from CBS systems versus from sewerage, and to characterize the full-cycle costs of excreta management under each approach. Such analysis could also explore the potential for revenue generation through waste processing and resource recovery. CBS may be more conducive to recovering energy and resources (e.g., phosphorus and nitrogen) from excreta, a practice that is increasingly promoted as a means of improving the sustainability of waste management.^(43)^ In contrast with most sewer systems, CBS provides source separation of waste streams and avoids diluting excreta with water. Source separation and concentrated material streams facilitate resource recovery at a lower unit cost than is possible using conventional treatment of mixed dilute streams.^(44)^ Moreover, it has also been suggested that material from CBS systems may have more embodied value than faecal sludge from on-site systems, because it is less decomposed and hence contains greater quantities of recoverable resources.^(45)^

In addition to cost, the political implications of network infrastructure development in unregularized communities may make CBS systems more attractive than piped sewer systems to urban planners in some settings. CBS systems require little or no permanent infrastructure installation in a community. As such, they give municipalities the flexibility of facilitating sanitation for urban residents without legitimizing illegal land development or making long-term investments in areas that are likely to undergo renewal or redevelopment.

For CBS systems to deliver on their potential benefits, they must be demonstrated to enable the hygienic isolation and management of human wastes. Earlier “bucket latrines” posed public health risks because of their poor toilet design and construction, spills, and other operational failures. Similarly, missed collections or failures to deliver sufficient quantities of Bonzodè during the pilot led some residents to revert to alternative sanitation habits or to dump waste in the environment, demonstrating how critical reliable operations are to the success of CBS systems. The two containers of waste that were reportedly dumped during the pilot represent less than 0.5 per cent of the faeces that were produced by household CBS users during the study period. Although quantitative comparisons are impossible, this mass is likely small in comparison with the amount of faeces mobilized by flooding from pit latrines and manual pit desludging that occurred during the same interval.

Appropriate planning for the frequency of waste collection and distribution of cover materials in CBS systems is paramount. The daily mass of faeces produced per capita can vary in different contexts, but the values observed in this study are consistent with those reported in literature.^(46)^ Local diets may influence the required frequency of collection. This aspect is particularly important with respect to cover material supply and its impact on collection logistics. Whereas Bonzodè constituted only 31 per cent of the mass of material collected during the pilot, it accounted for about 60–75 per cent of the volume. Cover material is thus the main driver of container fill rates and a substantial service cost driver. Future efforts in CBS system development would benefit from optimization of the cover material mix for system performance and efficiency. Future services might also experiment with “customer service outlets” in the community where households can obtain replacement containers or additional cover material when needed, a practice that occurred informally and to a limited extent during this study.

An additional concern for CBS services is preserving the dignity and social status of service operators. The labourers who provide manual emptying services for pit, dry and “bucket” latrines have historically suffered social stigma or ostracism.^(47)^ In Haiti, this stigma is driven by community perceptions of filth and contamination.^(48)^ The containers used for CBS service delivery prevent staff from coming into contact with excreta. Efforts were also made during the Shada pilot to ensure that staff had clean uniforms and personal protective equipment at all times. Anecdotal evidence suggests that these measures, along with operators’ use of equipment such as smartphones, conferred a professional aura upon the service that is respected by residents. Similar effects of investments in uniforms and access to technology have been shown with water and sanitation service providers in South Asia.^(49)^

Several other service and hardware design elements are important when considering the applicability of CBS in different contexts. For example, in Shada the CBS service did not manage households’ urine. This practice was acceptable in this setting and at pilot scale, but would likely present challenges elsewhere. Users may be unwilling to manage urine, or space, soil or groundwater constraints may make infiltration infeasible. Urine can also present public health risks from urine-borne pathogens or cross-contamination with faeces.^(50)^ CBS systems thus need context-appropriate urine management strategies, which could include collection by the service provider. Given the difficulty of transporting urine, it is likely that locating urine-processing facilities as close to users as possible would be desirable. The costs of such service changes would need to be carefully evaluated.

There are also important service and planning elements to consider in scaling up CBS services. For the forebears of modern CBS services, earth closets, maintaining a steady supply of cover material and sustaining proper use of the toilet were deemed persistent challenges.^(51)^ In the Shada service trial, cover material was indeed an important service cost driver, but appropriate toilet use was generally not a challenge. Both of these service aspects may become more complex at larger scale. More generally, earth closets were largely abandoned in the early 20th century because water closets (WCs) were perceived as more convenient, water was relatively abundant, and WCs easily channelled excreta and wastewater “away”.^(52)^ Despite their potential to improve excreta management in dense low-income areas, CBS systems face an enduring shortcoming relative to piped sewers in that they provide no solution for managing greywater (used for bathing or washing, rather than for excreta disposal).^(53)^ CBS systems may thus be an interim solution that is phased out once waterborne sewerage is widely practical. Alternatively, they may present an opportunity to rethink urban waste management planning such that high-concentration household residues like faeces and solid waste are handled by a “solid” collection system and low-concentration residues like greywater are conveyed in a “liquid” system.

For broad implementation, the CBS service would also need to be adapted to different cultural norms for toilet use and anal cleansing. In Shada, users preferred pedestal-style toilets for in-home use, and wiping for anal cleansing. Whereas the toilets in this service could be modified for squatting use with little difficulty, an entirely different design would likely be needed to accommodate anal washing. Other aspects that merit consideration when evaluating the applicability of CBS systems in different contexts include user acceptance of and willingness to pay for such services; alternative service models, such as subscription-based private enterprises or municipal service provision; appropriate regulatory models, perhaps inspired by frameworks for solid or hazardous waste management; and whether CBS systems are viewed as a short-, medium- or long-term sanitation service solution. Further work is also needed to develop cost estimates for CBS services at large scale, and to compare those costs against user willingness to pay and potential subsidies. An article by Russel et al., forthcoming in the October 2015 issue of this journal, will include findings from the Haiti pilot study pertaining to many of these aspects.

## Footnotes

2. International Monetary Fund (IMF) (n.d.), “IMF DataMapper”, accessed March 2012 at http://www.imf.org/external/datamapper/; also Joint Monitoring Programme (JMP) (2014), *Progress on Drinking Water and Sanitation: 2014 Update*, WHO and UNICEF, 28 pages.

3. See reference 2, Joint Monitoring Programme (JMP) (2014).

4. United Nations Economic & Social Affairs (UN-ESA) (2012), *World Urbanization Prospects: The 2011 Revision*, 50 pages.

5. Department of Water and Sanitation in Developing Countries/Swiss Federal Institute of Aquatic Science and Technology (Sandec/Eawag) (2006), “Urban Excreta Management - Situation, Challenges, and Promising Solutions”, 15 pages; also Koné, D (2010), “Making urban excreta and wastewater management contribute to cities’ economic development: a paradigm shift”, *Water Policy* Vol 12, No 4, pages 602–610.

6. Isunju, J B, K Schwartz, M A Schouten, W P Johnson and M P van Dijk (2011), “Socio-economic aspects of improved sanitation in slums: A review”, *Public Health* Vol 125, No 6, pages 368–376.

7. McFarlane, C (2008), “Sanitation in Mumbai’s informal settlements: state, ‘slum’, and infrastructure”, *Environment and Planning A* Vol 40, No 1, pages 88–107.

8. Based on linear regressions of JMP country files for urban sewer connections. Joint Monitoring Programme (n.d.), “JMP Country Files”, accessed June 2014 at http://www.wssinfo.org/documents/?tx_displaycontroller%5Btype%5D=country_files. (Note that data used for these estimates are aggregated from various different survey instruments and rely on respondents’ having accurate knowledge of where waste from their toilet goes. In this particular case, it was not practical to disaggregate shared sewer connections from private ones.)

9. See reference 5, Koné (2010); also Strauss, M, S A Larmie and U Heinss (2000), “Treating faecal sludges in ponds”, *Water Science & Technology* Vol 42, No 10–11, pages 283–290.

10. UN-Habitat (2003), “Rental housing: An essential option for the urban poor in developing countries”.

11. Jenkins, M and S Sugden (2006), “Rethinking sanitation: Lessons and innovation for sustainability and success in the new millennium”, UNDP Human Development Report Office background paper; also Wegelin-Schuringa, M and T Kodo (1997), “Tenancy and sanitation provision in informal settlements in Nairobi: revisiting the public latrine option”, *Environment and Urbanization* Vol 9, No 2, pages 181–190; and Adubofour, K, K Obiri-Danso and C Quansah (2013), “Sanitation survey of two urban slum Muslim communities in the Kumasi metropolis, Ghana”, *Environment and Urbanization* Vol 25, No 1, pages 189–207.

12. Jenkins, M and V Curtis (2005), “Achieving the ‘good life’: Why some people want latrines in rural Benin”, *Social Science & Medicine* Vol 61, No 11, pages 2446–2459.

13. See reference 11, Wegelin-Schuringa and Kodo (1997); also Schouten, M A and R W Mathenge (2010), “Communal sanitation alternatives for slums: A case study of Kibera, Kenya”, *Physics and Chemistry of the Earth* Vol 35, Nos 13–14, pages 815–822.

14. See reference 11, Jenkins and Sugden (2006); also Günther, I, C Niwagaba, C Lüthi, A Horst, H-J Mosler and I Tumbewaze (2012), “When is shared sanitation improved sanitation? - The correlation between number of users and toilet hygiene”, Urban Affordable Clean Toilets (U-ACT), 5 pages; and Heijnen M, O Cumming, R Peletz, G K-S Chan, J Brown, K Baker and T Clasen (2014), “Shared Sanitation versus Individual Household Latrines: A Systematic Review of Health Outcomes”, *PLoS ONE* Vol 9, No 4, page e93300.

15. Cairncross, S (1992), “Sanitation and water supply: practical lessons from the decade”, World Bank Water and Sanitation Discussion Paper Series Vol 9; also Joshi, D, B Fawcett and F Mannan (2011), “Health, hygiene and appropriate sanitation: experiences and perceptions of the urban poor”, *Environment and Urbanization* Vol 23, No 1, pages 91–111.

16. See reference 6.

17. See reference 15, Cairncross (1992).

18. Based on 2.4 million urban residents using on-site systems and producing 1 litre/capita/day of faecal sludge. Koné, D, O Cofie and K L Nelson (2010), “Low-Cost Options for Pathogen Reduction and Nutrient Recovery from Faecal Sludge”, in P Drechsel, C Scott, L Raschid-Sally, M Redwood and A Bahri (editors), *Wastewater Irrigation and Health: Assessing and Mitigating Risk in Low-income Countries*, Earthscan, London, pages 1–18.

19. See reference 5, Department of Water and Sanitation in Developing Countries/Swiss Federal Institute of Aquatic Science and Technology (Sandec/Eawag) (2006); also Parkinson, J and M Quader (2008), “The challenge of servicing on-site sanitation in dense urban areas: Experiences from a pilot project in Dhaka”, *Waterlines* Vol 27, No 2, pages 149–163; also Ingallinella, A M, G Sanguinetti and T Koottatep (2002), “The challenge of faecal sludge management in urban areas- strategies, regulations and treatment options”, *Water Science & Technology* Vol 46, No 10, pages 285–294; Rydin, Y, A Bleahu, M Davies, J D Dávila, S Friel, G De Grandis, N Groce et al. (2012), “Shaping cities for health: complexity and the planning of urban environments in the 21st century”, *The Lancet* Vol 379, No 9831, pages 2079–2108; and Khan, M S, J Fisher and A Nkansah (2012), “Manual pit emptying as a sustainable livelihood in Ghana”, *Proceedings of the ICE - Engineering Sustainability* Vol 165, No 3, pages 215–221.

20. Newsholme, A (1902), *Hygiene: a manual of personal and public health*, George Gill & Sons LD, London, 372 pages.

21. See reference 15, Cairncross (1992); also Van Der Geest, S (2002), “The night-soil collector: Bucket latrines in Ghana”, *Postcolonial Studies* Vol 5, No 2, pages 197–206.

22. Nilsson, D (2006), “A heritage of unsustainability? Reviewing the origin of the large-scale water and sanitation system in Kampala, Uganda”, *Environment and Urbanization* Vol 18, No 2, pages 369–385.

23. Letema S, B van Vliet and J B Van Lier (2014), “Sanitation policy and spatial planning in urban East Africa: Diverging sanitation spaces and actor arrangements in Kampala and Kisumu”, *Cities* Vol 36, pages 1–9, page 3.

24. See reference 11, Adubofour et al. (2013).

25. Moule, H and H Girdlestone (1870), “Improvement in earth-closets”, U.S. Patent No. 4138, Google Patents.

26. Waring, G E (1870), “Earth-Closets and Earth Sewage”, The Tribune Association, New York.

27. In thermophilic composting, biological waste is broken down by heat-tolerant bacteria. The high temperatures produced by the decomposition de-activate pathogens in the waste. Preneta, N, S Kramer, B Magloire and J M Noel (2013), “Thermophilic co-composting of human wastes in Haiti”, *Journal of Water, Sanitation and Hygiene for Development* Vol 3, No 4, pages 649–654.

28. User-centred design, often also referred to as human-centred design, is a design process that relies on extensive user interviews and iterative prototyping to develop products or services that meet the needs and aspirations of target users. IDEO (2009), “Human-Centered Design Toolkit”, 105 pages.

29. Respondents indicated using the option during the morning, afternoon, evening or night. “Evening” and “Night” were considered nighttime use. “Morning” was categorized as daytime use, even though it includes pre-dawn defecation.

30. Only the adult stalls, which have urine separation, are used in this calculation. Since the child stalls mix faeces and urine, it was not possible to isolate the mass of faeces from the mass of urine.

31. Other disposal alternatives including the street, canals, river, pit latrines, burial, or trash heaps were not considered safe management.

32. Whereas information was collected at the household level, the analyses presented in this study were carried out at the cohort level.

33. A “flying toilet” is a plastic bag that is used to collect faeces during in-home defecation and is then thrown into an alley, a waterway, solid waste bin or rooftop.

34. Specifically, the share of the control cohort that reported using their neighbour’s pit latrine increased from 2 per cent to 9 per cent between baseline and endline.

35. Total usage was calculated using data from 13 of the 15 observation days, because data for the children’s stall on two days were lost through hardware failures.

36. One day of mass measurements was lost due to a hardware failure during data download.

37. This estimate assumes that sanitation practices of the five treatment cohort households that could not be interviewed at endline are similar to those reported by interviewed households.

38. Although 132 CBS toilets were deployed, 110 households reported regularly using them, yielding 220 expected collections per week with a linear ramp-up in the first four weeks of service.

39. It is unknown what recourse they took to defecate in the interim.

40. Condominial sewerage is simplified sewerage designed with the consultation of property owners and individual users. This process often results in shorter sewers that are located within property blocks instead of in the public right of way.

41. Mara, D D (1996), *Low-cost sewerage*, Wiley, Chichester, UK, 14 pages; also Nance, E and L Ortolano (2007), “Community Participation in Urban Sanitation: Experiences in Northeastern Brazil”, *Journal of Planning Education and Research* Vol 26, No 3, pages 284–300; Sinnatamby, G S (1983), “Low-cost sanitation systems for urban peripheral areas in North-east Brazil”, PhD thesis, University of Leeds; Sinnatamby, G S, D D Mara and M McGarry (1986), “Sewerage: shallow systems offer hope to slums”, *World Water* Vol 9, pages 39–41; Sinnatamby, G S No 1, (1986), “The design of shallow sewer systems”, United Nations Centre for Human Settlements, Nairobi; Kalbermatten J, D Julius, C Gunnerson and D D Mara (1982), *Appropriate Sanitation Alternatives: A Planning and Design Manual*, Johns Hopkins University Press, Baltimore, 176 pages; and Hutton, G (2004), “Evaluation of the Costs and Benefits of Water and Sanitation Improvements at the Global Level”, World Health Organization, Geneva.

42. See reference 41, Hutton (2004).

43. Muspratt, A M and I Ray (2010), “Commentary: Back-End Users: The Unrecognized Stakeholders in Demand-Driven Sanitation”, *Journal of Planning Education and Research* Vol 30, No 1, pages 94–102.

44. Zeeman, G, K Kujawa-Roeleveld, T De Mes, L Hernandez, M De Graaff, L Abu-Ghunmi, A Mels et al. (2008), “Anaerobic treatment as a core technology for energy, nutrients and water recovery from source-separated domestic waste(water)”, *Water Science & Technology* Vol 57, No 8, pages 1207–1212; also Zeeman, G and K Kujawa-Roeleveld (2011), “Resource recovery from source separated domestic waste(water) streams; full scale results”, *Water Science …Technology* Vol 64, No 10, pages 1987–1992; and Langergraber, G and E Muellegger (2005), “Ecological Sanitation—a way to solve global sanitation problems?”, *Environment International* Vol 31, No 3, pages 433–444.

45. See reference 19, Ingallinella et al. (2002).

46. Kujawa-Roeleveld, K (2001), “Types, characteristics and quantities of domestic solid waste”, in P Lens, G Zeeman and G Lettinga (editors), *Decentralised Sanitation and Reuse: Concepts,Systems, and Implementation*, International Water Association, London, pages 73–93; also Mara, D D (2004), *Domestic wastewater treatment in developing countries*, Earthscan, London, 310 pages; Kujawa-Roeleveld, K, T Elmitwalli, A Gaillard, M Van Leeuwen and G Zeeman (2003), “Co-digestion of concentrated black water and kitchen refuse in an accumulation system within the DESAR (decentralized sanitation and reuse) concept”, *Water Science & Technology* Vol 48, No 4, pages 121–128; and Mang H-P and Z Li (2010), “Technology review of biogas sanitation”, GIZ, pages 1–21.

47. See reference 15, Cairncross (1992).

48. Neiburg, F and N Nicaise (2011), *Garbage. Stigmatization, Commerce, Politics (Déchets.Stigmatisations, Commerces, Politiques)*, Interuniversity Institute for Research and Development, 123 pages.

49. Davis, J (2004), “Corruption in public service delivery: experience from South Asia’s water and sanitation sector”, *World Development* Vol 32, No 1, pages 53–71.

50. Höglund, C (2001), “Evaluation of microbial health risks associated with the reuse of source-separated human urine”, PhD thesis, Swedish Institute for Infectious Disease Control, 87 pages.

51. See reference 20.

52. See reference 20.

53. See reference 20.
